# A Novel Motion Platform Based on Dual Driving Feet Linear Ultrasonic Motor

**DOI:** 10.3390/mi16091039

**Published:** 2025-09-10

**Authors:** Yue Jian, Zhen Liu, Ping Yao, Wenjie Zhou, Junfeng He, Huazhuo Liang

**Affiliations:** 1 School of Mechatronic Engineering, Guangdong Polytechnic Normal University, Guangzhou 510665, China; jianyue@gpnu.edu.cn (Y.J.); ypsunny@163.com (P.Y.); zhouwj1001@163.com (W.Z.); hejunfeng@gpnu.edu.cn (J.H.); lianghuazhuo@gpnu.edu.cn (H.L.); 2 Jihua Laboratory, No. 28 Island Ring South Road, Guicheng Street, Nanhai Distrct, Foshan 528200, China

**Keywords:** linear ultrasonic motor, motion platform, preload device, high-precision

## Abstract

This paper presents a novel motion platform based on a π-shaped linear ultrasonic motor. Initially, a new preload device was designed in accordance with established criteria for high-power linear ultrasonic motors. Mounted on the base structure, this mechanism neither interferes with the stator’s high-frequency vibrations nor couples with the mover’s motion. Structural parameters were determined through theoretical modeling, while experimental validation confirmed the mechanism’s capability to deliver stable and appropriate preload throughout the motor’s complete operating cycle, thereby enhancing the platform’s operational stability and positioning accuracy. Subsequently, a novel mover was developed by replacing linear guides with a ceramic–ceramic mechanism. This mover features a compact structure and flexible design, facilitating both miniaturization and effective stroke amplification. The resulting platform achieves a 40% reduction in volume compared to conventional designs while extending the stroke to 150% of the original capacity. Finally, a prototype was fabricated and experimentally evaluated. Test results demonstrate output velocities exceeding 200 mm/s in both directions, with positioning accuracy reaching 1.1 μm.

## 1. Introduction

Long-stroke, high-precision, and high-resolution motion platforms hold an exceptionally significant position within modern industrial production and scientific research [1,2,3,4]. The positioning accuracy and effective stroke of such platforms directly impact the precision and scope of manufacturing and processing operations. Furthermore, the driving elements of the platform fundamentally determine its response speed, operating velocity, accuracy, and achievable stroke range. Linear ultrasonic motors (LUMs) are characterized by their rapid response, high positioning accuracy, and the inherent capability to generate direct linear motion without requiring transmission mechanisms [5,6,7,8]. Consequently, positioning platforms driven by LUMs offer distinct advantages, including a short transmission chain, large stroke, and high positioning accuracy [9,10].

Since their emergence in the 1940s, ultrasonic motors (USMs) have garnered extensive attention from researchers due to their unique operating principles and superior electromechanical performance. In 1948, Williams and Brown designed the first prototype USM, elucidated its fundamental working mechanism, and filed the first patent for a “piezoelectric motor”. This marked the inception of a surge in USM research, leading to the continuous emergence of various compact, high-precision and high-output USM configurations [11,12,13,14,15]. In 1998, Japanese researchers Kurosawa et al. proposed a high-speed, high-thrust linear ultrasonic motor (LUM) [16]. This motor employed a stator design combining dual longitudinal vibration modes, composed of two sets of Langevin vibrators. By exciting symmetric and antisymmetric modes within the stator, elliptical motion trajectories were formed on the driving feet surfaces of the vibrators. Experimental results demonstrated a maximum no-load speed of 3.5 m/s and a maximum thrust of 51 N for this motor. Owing to its excellent mechanical output performance, this V-shaped LUM has been extensively researched and applied [17,18,19,20]. For instance, Asumi et al. [19,20] achieved miniaturization of the V-shaped motor; the scaled-down version attained a maximum output speed of 1.5 m/s and a maximum thrust of 25 N and employed an inertia drive mechanism to achieve low-speed operation. In 2006, Zhang developed a miniature tubular piezoelectric ultrasonic motor utilizing a PZT-metal composite stator (1.0 mm OD × 5.0 mm length) and dual steel rotors. The motor operates at 58 kHz resonance in its first circular-bending mode, driven by two 90° phase-shifted alternating voltage pairs, achieving high rotational speeds exceeding 3000 rpm and a starting torque of 7.8 μN·m. With an assembled diameter of 1.0 mm and length of 8 mm, this design demonstrates suitability for confined micro-scale applications such as intravascular devices and microrobotics [21]. In 2011, Japanese researcher T. Masahiro [22] developed a multilayer transducer-structured LUSM utilizing an independent electrode array. This motor featured a compact structure (dimensions: 30 mm × 8.4 mm × 4 mm), exhibited good controllability, achieved a positioning resolution of 20 nm, and could operate at low speeds down to 0.1 mm/s. In 2020, Xin et al. from Peking University designed a single-mode LUM employing a circular ring structure [23]. This novel motor delivered a maximum output force of 2.7 N and a no-load speed of 56 mm/s, while maintaining a high positioning accuracy of 0.1 μm.

A comprehensive analysis of the aforementioned research landscape reveals that USMs possess the inherent advantages of flexible design and ease of miniaturization. These advantages enable their configuration to be readily adapted to meet the specific requirements of confined spaces or specialized application scenarios [24,25]. Concurrently, USMs exhibit micrometer/nanometer-level positioning capabilities, endowing them with significant potential for development within the field of micro-manipulation [26,27]. Furthermore, these motors feature an inherently compact overall structure with a minimal number of components [28,29]. Crucially, they can provide speed reduction without requiring complex transmission mechanisms, and they inherently provide automatic self-locking upon power-off without necessitating a separate locking mechanism. Consequently, employing USMs to construct the drive system for motion platforms holds considerable promise for substantially reducing the volume and weight of the drive mechanism while simultaneously enhancing the positioning accuracy of the platforms. However, the current limitations in the output characteristics of existing USMs constrain their broader application in motion platforms, as it remains challenging to simultaneously achieve a large stroke, compact size, high output, and high positioning accuracy.

**Figure 1:**
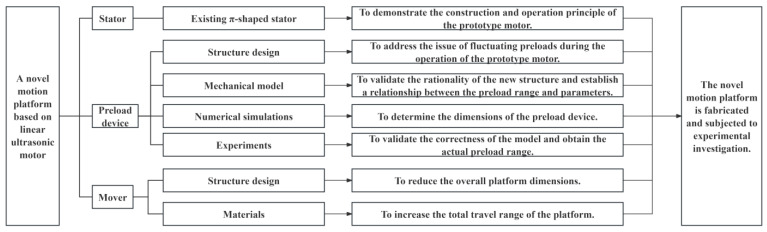
Flowchart of the present study.

To address these challenges, this paper proposes a novel motion platform based on a π-shaped stator. Firstly, the structure and operational principle of an existing motion platform based on a π-shaped stator are elucidated. Subsequently, to address the issue of varying preload during operation in conventional platforms, a novel preload device is proposed. This device is mounted on the base, thereby remaining detached from the high-frequency vibrating stator and stationary relative to the mover’s motion. A mechanical model of the new preload device is established, and its specific dimensions are determined through numerical simulation. The actual preload range is subsequently obtained via experimental measurement. Furthermore, the mover structure of the motion platform is redesigned. Commercial guides are replaced with ceramic bars to construct the platform’s mover, resulting in a more compact form factor and an extended travel range. Finally, a prototype of the novel motion platform was manufactured and assembled, and its output performance was experimentally evaluated. A detailed step-by-step flowchart of the present study is provided in Figure 1.

## 2. Structure and Principle of the Platform

**Figure 2:**
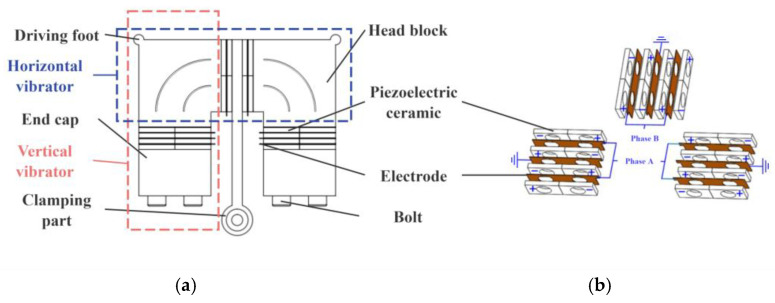
Specific view of the stator. (**a**) Stator component configuration. (**b**) Layout of piezoelectric ceramics.

Plate-type linear ultrasonic motors are well-suited as drive units for motion platforms with confined installation space due to their inherent flat profile, compact design, and ease of miniaturization. However, the vibration mode distribution within such plate-type structures under multi-modal excitation is complex, posing significant challenges in identifying suitable clamping locations. If a single-mode excitation strategy is adopted, decoupling the complex operating modes of the plate structure into simpler, low-order vibration modes with clearly identifiable antinode points offers a promising solution to the clamping issue. Consequently, this project preliminarily selects a π-shaped stator configuration (as illustrated in Figure 2).

Figure 2a illustrates the schematic of the π-shaped stator structure [30]. Overall, the stator constitutes an axisymmetric π-shaped plate structure. Two driving feet are positioned at the vertices on either side of the stator, while the clamping section is located centrally. The stator consists of head blocks, piezoelectric ceramics, end caps, a clamping part, electrodes, and bolts. The piezoelectric ceramics are clamped between elastic bodies via tightening bolts. To elucidate the motor’s operating principle, the rectangular vibrator assembly formed by the sequential arrangement of a head block, piezoelectric ceramics, and another head block along the horizontal direction is termed the horizontal vibrator. Conversely, the rectangular vibrator assembly formed by the sequential arrangement of a head block, piezoelectric ceramics, and an end cap along the vertical direction is termed the vertical vibrator. Consequently, the π-shaped stator can be conceptualized as being formed by the cross-coupling of two vertical vibrators and one horizontal vibrator assembly.

The arrangement of piezoelectric ceramics within the stator is depicted in Figure 2b. A total of 24 piezoelectric ceramic plates are employed. These plates are grouped into 12 sets, with each set comprising two adjacent plates possessing opposite polarization directions. Four sets of piezoelectric ceramic plates are installed within each vibrator. Each pair of adjacent sets is symmetrically arranged about the electrodes (or clamping part) sandwiched between them. The two electrodes within the horizontal vibrator are interconnected by a conductor. In each vertical vibrator, the electrodes on both sides are connected by conductors, and the central electrode is grounded. The head blocks, end caps, and clamping part are also grounded. All piezoelectric ceramics in the stator are divided into two phases: those placed within the vertical vibrators are designated as Phase A, while those within the horizontal vibrator are designated as Phase B. Consequently, when excitation signals are applied to the Phase A and Phase B ceramic plates, the piezoelectric ceramic plates on one side within each vibrator assembly expand simultaneously, while those on the opposite side contract simultaneously, and vice versa.

**Figure 3:**
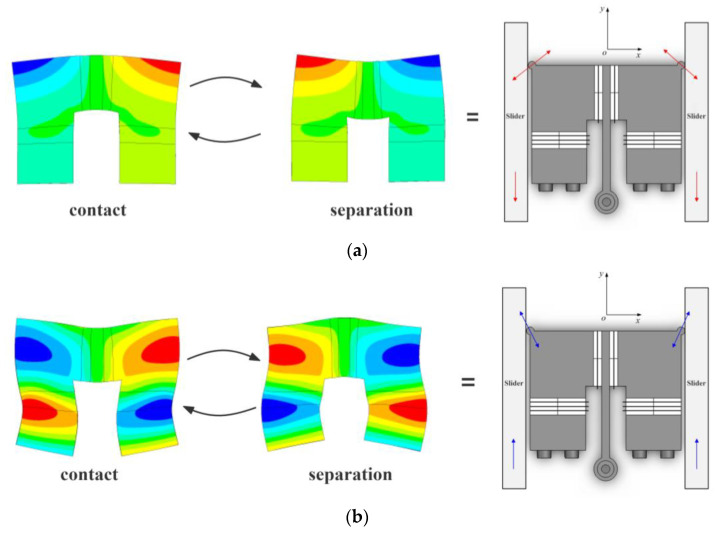
Operating principle of stator. (**a**) Retraction mode. (**b**) Extension mode.

The π-shaped stator employs a single-mode excitation strategy, enabling it to utilize distinct operational modes for motion in two opposing directions. The specific operating principle of the motor is illustrated in Figure 3.

When sinusoidal excitation signals of identical frequency but with a 180-degree phase difference are applied to the Phase A and Phase B piezoelectric ceramic plates, the stator is excited into its operational mode shown in Figure 3a, termed the retraction mode. In this mode, while the horizontal vibrator contracts outward and expands inward (or conversely, expands outward and contracts inward), the vertical vibrators simultaneously exhibit the opposite behavior: expanding outward and contracting inward (or contracting outward and expanding inward). Within one excitation cycle, two distinct states exist between the stator and mover: contact and separation. During the contact state, the stator presses against the mover, generating a force component in the negative y-direction. This force propels the mover along the negative y-axis. Subsequently, the stator and mover enter the separation state, wherein the stator no longer drives the mover. The mover continues its motion in the negative y-direction due to inertia. This cyclic process enables the mover to execute continuous retracting motion.

Conversely, when in-phase sinusoidal excitation signals of identical frequency are applied to the Phase A and Phase B piezoelectric ceramic plates, both the horizontal and vertical vibrators simultaneously expand outward and contract inward (or contract outward and expand inward), as shown in Figure 3b. This operational mode is termed the extension mode of the stator. Under this mode, the mover is propelled along the positive y-axis, executing the extension motion. By adjusting the frequency and phase difference in the two excitation signals, the motor can switch between these two operational modes, thereby reversing its direction of motion.

**Figure 4:**
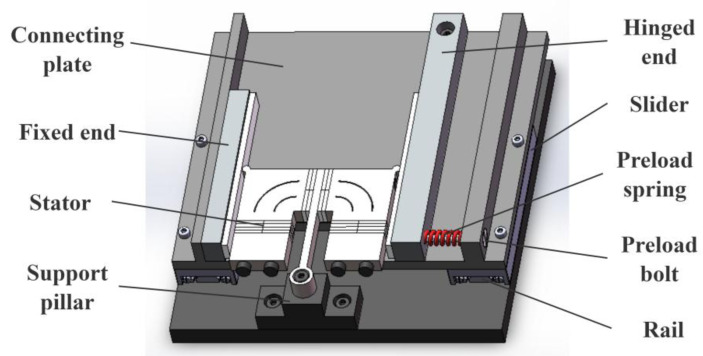
Configuration of the motor.

A classic motion platform structure based on the π-shaped motor is illustrated in Figure 4. The overall assembly comprises the stator, mover, preload device, and base. A support pillar is mounted on the base to uphold the stator’s clamping element, suspending the stator and ensuring contact with the mover occurs exclusively at the driving feet. Two linear guides serve as the translational joints of the motion platform. Their rails are installed on opposite sides of the base, parallel to the long edges of the vertical vibrator within the stator. The sliders of these guides are connected via a flat plate, which, together with the sliders, constitutes the mover of the motor. The preload device consists of a fixed end, a hinged end, a preload spring, and a preload bolt. The mover components contacting the stator’s driving feet are designated as the fixed end and the hinged end. The fixed end is rigidly attached to the connecting plate and is fitted with a friction ceramic strip, contacting one driving foot of the stator. Conversely, the hinged end is pivotally connected to the connecting plate via a bolt and is also fitted with a friction ceramic strip, contacting the stator’s other driving foot. Prior to motor operation, tightening the bolt compresses the spring, generating a preload force between the hinged end and the stator. At this stage, the stator can undergo slight rotation about its clamping element, equalizing the preload magnitude at both driving feet. The preload force between the stator and mover can be adjusted by selecting springs of different stiffnesses and varying the spring compression amount. During motor operation, the stator propels the mover along both positive and negative y-axis directions.

While this motion platform enables bidirectional mover movement, it exhibits inherent limitations. Firstly, the current preload application method is unstable. During motor operation, the preload device moves continuously with the motor’s mover. This causes the distance between the preload application point and the stator’s driving feet to vary constantly with the relative position of the stator and mover, thereby inducing instability in the preload force during operation. This instability ultimately degrades the motor’s output performance and operational stability. Secondly, the mover assembly inherently includes the sliders, connecting plate, and preload device, resulting in a bulky and heavy overall structure. This substantially reduces the platform’s effective load capacity. Finally, commercially available linear guides, such as the VRT 2095A (SELN, Dongguan, China) model referenced in this work, possess fixed dimensions and stroke relative to their size. For instance, the VRT 2095A has a maximum stroke of 60 mm, yet its actual length is 95 mm. This characteristic impedes platform miniaturization.

To address the aforementioned issues, the following sections will present optimized designs focusing on both the preload device and the mover structure. The goal is to construct a motion platform featuring a more compact structure, reduced footprint, enhanced design flexibility, and improved operational stability.

## 3. Design of Preload Device

### 3.1. Structure and Principle of Preload Device

Stable preload maintenance within an appropriate range is essential for ensuring reliable operation of LUMs. An unstable preload state degrades operational stability and induces energy dissipation, while an unsuitable preload range prevents the motor from achieving optimal performance or potentially leads to operational failure. Conventionally, preload devices for LUMs are integrated directly onto the stator, utilizing elastic components such as springs, disc springs, or flexible hinges to apply force. However, the stator’s inherent high-frequency vibration during operation inevitably compromises the stability of these elastic elements. This leads to significant thermal energy dissipation and adversely impacts preload stability, consequently degrading motor output performance and operational reliability.

**Figure 5:**
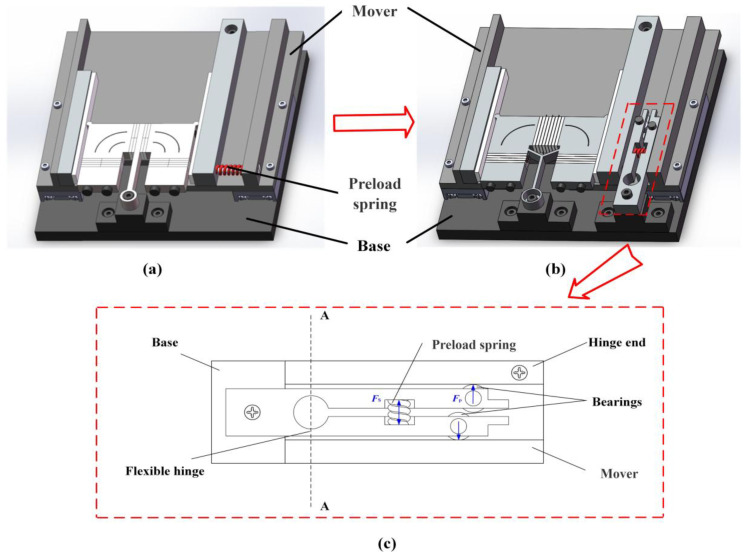
Preload device for (**a**) original and (**b**) novel motion motor. (**c**) Specific view of novel preload device.

Reference [31] investigates the influence of preload device stiffness—specifically tangential and normal components—on motor output characteristics through modeling and experimentation. The study demonstrates that appropriate normal stiffness enhances motor stability while preserving unimpeded stator vibration. Conversely, increased tangential stiffness correlates positively with improved output characteristics. To meet these stiffness requirements, a one-hinge-end preload application method was developed for the π-shaped motor (Figure 5a). Experimental validation confirms this method enhances motor output performance. Critically, isolating the preload device from the stator mitigates adverse effects of stator vibration on preload stability, reducing thermal losses and energy dissipation.

Nevertheless, mounting the preload device on the mover introduces a significant limitation: the dynamic displacement between the driving foot and preload application point during operation induces continuous preload variation. This fluctuation detrimentally affects motor output characteristics and stability.

Consequently, a novel preload device is proposed (Figure 5b,c), designed to replace the original preload spring. Mounted rigidly on the base, this mechanism adopts a clamped configuration featuring a closed bottom and bifurcated upper arms. Precision bearings are integrated laterally onto these forked arms, establishing contact interfaces with both the hinged stator end and the mover. Critically, the bearing–hinge contact point and driving foot–hinge interface are horizontally collinear. The device incorporates a central groove for preload spring installation. By compressing the spring (whose free length exceeds the groove width) during assembly, a radial expansion force *F*s is generated. This force drives the bifurcated arms apart, inducing a normal contact force *F*_p_ at the bearing–hinge interface. Consequently, an equivalent preload *F*_p_ is transmitted to the hinge–driving foot contact point. Under this loading, the stator undergoes minor rotation about its hinge to equilibrate preload forces across both driving feet, ultimately yielding a total motor preload of 2 *F*_p_.

### 3.2. Model and Dimension of Preload Device

**Figure 6:**
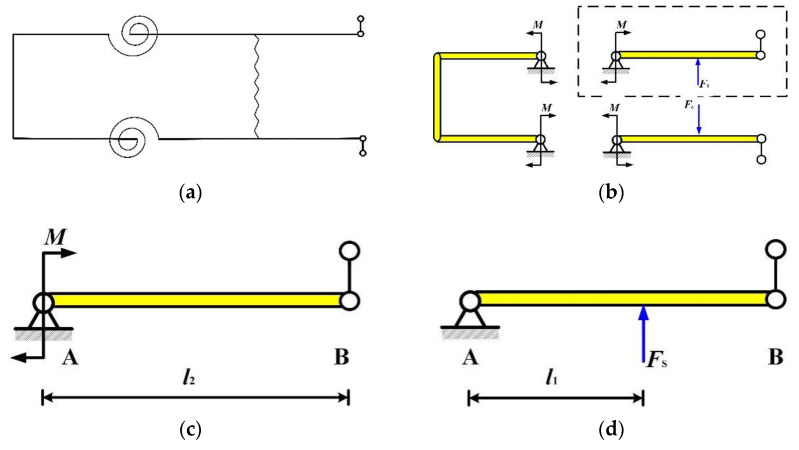
Mechanical model of preload device (**a**) and simplified model (**b**). Specific two statically determinate systems (**c**,**d**).

To determine the appropriate preload range, a mechanical model of the preload device was established, as illustrated in Figure 6. By simplifying the flexure hinge component as a torsion spring, the mechanical model shown in Figure 6b was derived. This model was sectioned along plane A-A (refer to Figure 6c), and the upper-right section was selected for analysis. The resulting system is statically indeterminate to the first degree, which can be decomposed into the superposition of two statically determinate systems, as depicted in Figure 6c,d.

Let the preload spring stiffness be *K*_0_ and its compression be *ε*. Consequently, the *φ* force exerted by the preload spring is

(1) $$F_{s}=K_{0}\varepsilon$$

And let the flexure hinge’s stiffness be denoted by *K*_α_. The deformation at end A, *φ*_1_ can be expressed as

(2) $$\varphi_{1}=\frac{M}{K_{\alpha }}$$

For the hinged beam AB, the total deformation at end A results from the superposition of deformations induced by force $F_{s}$ and couple *M*, that is

(3) $$\phi_{1}=\phi_{1F}+\phi_{1M}$$

The deformations *φ*_1*F*_ and *φ*_1*M*_ are derived as follows:

(4) $$\phi_{1F}=-\frac{F_{s}l_{1}(2l_{2}^{2}-3l_{1}l_{2}+l_{1}^{2})}{6EIl_{2}}$$

(5) $$\phi_{1M}=\frac{l_{2}M}{3EI}$$

where *l*_2_ denotes the total length of beam AB, *l*_1_ represents the distance from the action point of *F*_S_ to end A, *E* is the elastic modulus, and *I* is the inertia moment of the beam.

The couple *M* can be reduced as

(6) $$M=\frac{F_{S}l_{1}\left(2l_{2}-3l_{1}+\frac{l_{1}^{2}}{l_{2}^{2}}\right)}{2l_{2}+\frac{6EI}{K_{\alpha }}}$$

From the moment balance condition,

(7) $$M+F_{P}l_{2}-F_{S}l_{1}=0$$

Finally, *F*_p_ can be reduced as

(8) $$F_{P}=\frac{F_{S}l_{1}}{l_{2}}\left[1-\frac{\left(l_{2}-l_{1}\right)\left(2l_{2}-l_{1}\right)}{2l_{2}\left(l_{2}+\frac{3EI}{K_{\alpha }}\right)}\right]=\frac{K_{0}\varepsilon l_{1}}{l_{2}}\left[1-\frac{\left(l_{2}-l_{1}\right)\left(2l_{2}-l_{1}\right)}{2l_{2}\left(l_{2}+\frac{3EI}{K_{\alpha }}\right)}\right]$$

Since *l*_2_ is a quantity dependent on the stroke and structural dimensions of motor, it is determined during the initial design phase when structural constraints are considered. As evident from Equation (8), when this preload device is employed, the magnitude of the preload force obtained by the motor is related to the stiffness *K*_0_ of the preload spring, the distance *l*_1_ from its placement location to the flexure hinge position, and the stiffness *K*_α_ of the flexure hinge. Specifically, the preload force magnitude increases with the stiffness *K*_0_ of the preload spring, increases with the distance *l*_1_ between the preload spring placement and the flexure hinge position, and decreases with the stiffness *K*_α_ of the flexure hinge.

During actual motor operation, the preload force must be maintained within an appropriate range. Failure to do so will prevent the motor from achieving its optimal operational state—or even functioning altogether—regardless of any adjustments made to the excitation signal. Furthermore, within this appropriate range, establishing the relationship between the preload force and the spring stiffness enables control of the preload force by replacing springs with different stiffness values. This, in turn, allows for regulation of the motor’s operational state, enabling it to cater to diverse application requirements.

For this novel preload device, once fabricated, the distance *l*_1_ between the placement position of the preload spring and the flexure hinge position, as well as the stiffness *K*_α_ of the flexure hinge itself, become fixed parameters. Consequently, to ensure the preload force consistently remains within the appropriate range and can be adjusted by varying the stiffness *K*_0_ of the preload spring—thereby enabling the motor to attain different operational states—it is essential to first determine the values of *l*_1_ and *K*_α_.

**Figure 7:**
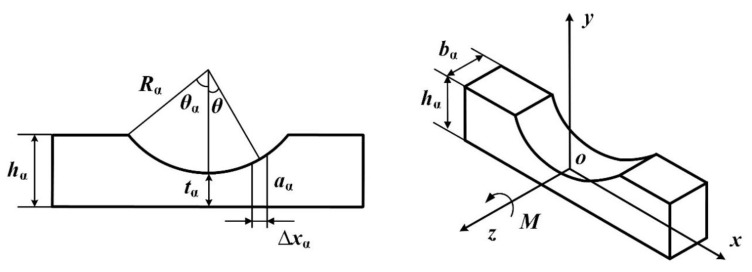
Unilateral arc flexure hinge.

Existing research [32] indicates that for the unilateral arc flexure hinge employed in this preload device (Figure 7), the primary influencing factors on its stiffness *K*_α_, listed in descending order of impact, are as follows: the minimum thickness *t*_α_, the width *b*_α_, and the radius *R*_α_ of the flexible arc. Let the height of the flexure hinge be denoted as *h*_α_ and its central angle as *θ*_α_.

If an infinitesimal segment along the *x*-axis is considered, with a height of *a*_α_ and a length of Δ*x*_α_, then the angular deformation *α* of the unilateral flexure hinge under the applied moment *M* is given by

(9) $$\alpha =\int_{-R_{\alpha }sin\theta_{\alpha }}^{R_{\alpha }sin\theta_{\alpha }}\frac{M_{z}\left(x\right)}{EI_{z}\left(x\right)}dx=\frac{12M}{Eb_{\alpha }R_{\alpha }^{2}}\int_{-\theta_{\alpha }}^{\theta_{\alpha }}\frac{\cos\theta }{{\left(\frac{t_{\alpha }}{R_{\alpha }}+2-2\cos\theta \right)}^{3}}d\theta$$

Let

(10) f=∫−θαθαcosθtαRα+2−2cosθ3dθ=8γ42γ+1tanθα24γ+121+4γ+1tan2θα22+4γ36γ3+3γ+1tanθα24γ+121+4γ+1tan2θα2+12γ42γ+14γ+152arctan4γ+1tanθα2

where

(11) $$\gamma =\frac{t_{\alpha }}{R_{\alpha }}$$

The stiffness *K*_α_ of the unilateral flexure hinge is expressed as

(12) $$K_{\alpha }=\frac{M}{\alpha }=\frac{Eb_{\alpha }R_{\alpha }^{2}}{12f}$$

As the novel preload device includes two unilateral arc flexure hinges, its stiffness should be expressed as

(13) $$K_{T}=2K_{\alpha }=\frac{Eb_{\alpha }R_{\alpha }^{2}}{6f}$$

**Figure 8:**
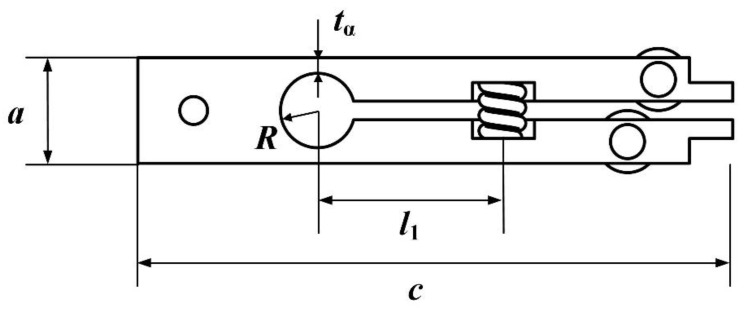
Dimension of preload device.

Incorporating the dimensions of the preload device illustrated in Figure 8, where the width, height, and length of the mechanism are denoted as *a*, *b*, and *c*, respectively, the following relationship holds:

(14) $$b=b_{\alpha }$$

(15) $$R=R_{\alpha }$$

(16) $$R+t_{\alpha }=\frac{a}{2}$$

Ultimately, the dimensions of the preload device are selected as a width *a* of 17 mm, a height *b* of 10 mm, and a length *c* of 96 mm. This selection integrates the dimensional requirements of the π-shaped motor while comprehensively considering overall system volume and manufacturability constraints. Through geometric relationships, the range for *l*_1_ is determined to be 12 mm to 30 mm, and for *R*, 2 mm to 6 mm. Given that readily available commercialized springs’ stiffness values range from 15.6 N/mm to 166.5 N/mm, and with the spring compression set at 1 mm, the resulting force *F*_s_ varies between 15.6 N and 166.5 N. Based on prior experimental evidence [31], linear ultrasonic motors achieve optimal performance when the preload force falls within the range of 60 N to 200 N. The novel preload device is fabricated from Grade 45 steel, which exhibits a Young’s modulus of 210 GPa. Numerical simulations were subsequently employed to finalize the dimensions of both the preload device and the flexure hinge structure. To facilitate machining and assembly, dimensional parameters were rounded to practical values. The finalized dimensional parameters of the preload device are summarized in Table 1.

Under these structural parameters, the simulated range of the preload force *F*_p_ is 12.2 N to 130 N. Given that the linear ultrasonic motor employs a dual driving feet configuration, the actual preload force experienced by the motor is twice *F*_p_, resulting in an effective preload range of 24.4 N to 260 N. This satisfies the validated range requirements for the π-shaped motor’s operation established through experimentation. Subsequently, with the dimensions of the preload structure finalized, the preload force applied to the motor can be controlled by utilizing preload springs of varying stiffness. This capability provides a direct means to precisely regulate the motor’s output characteristics, thereby making it adaptable to a wide range of operational demands.

### 3.3. Experiments on Preload Device

**Figure 9:**
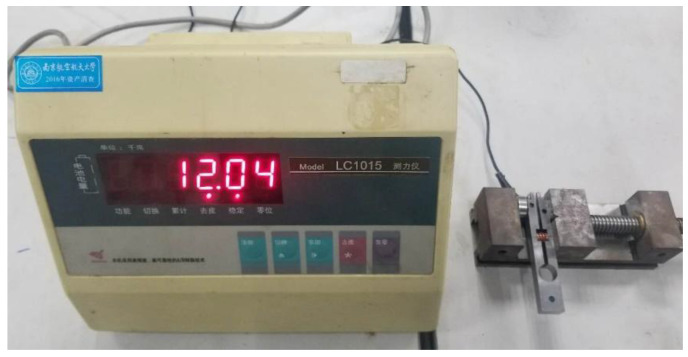
Experimental setup for preload device.

To validate the correctness of the preload model and assess the appropriateness of the preload range, a prototype of the novel preload device was fabricated. The achievable preload range provided by this mechanism was then experimentally measured using the setup depicted in Figure 9. A preload spring was installed within the fabricated prototype. The compressed spring induces slight deflection at the open end of the prototype. This deflected end, along with a force sensor probe, was secured in a bench vise. The vise was tightened until the open end returned to its undeflected position. At this state, the force *F*_P_ displayed on the force gauge corresponds to the force generated at the open end due to spring compression. Consequently, the preload force attainable by the motor is twice *F*_P_. The process was repeated by replacing springs of varying stiffnesses, and the corresponding *F*_P_ values were recorded via the force gauge.

**Figure 10:**
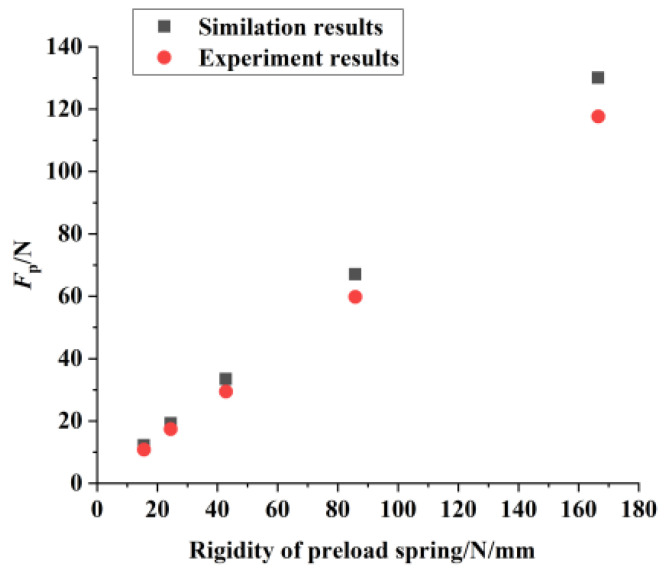
Experimental results and simulation results of preload.

A comparison of the experimental and simulation results is presented in Figure 10. The experimentally measured *F*_P_ range was 10.8 N to 117.6 N, corresponding to a total motor preload range of 21.6 N to 235.2 N. The discrepancy between the experimental and simulation results was within 12%, primarily attributed to differences between the material property values used in the simulation and those of the actual components, as well as tolerances introduced during machining and assembly. These results indicate that the mechanical model of the preload device provides a reasonably accurate prediction of the preload range. It should be noted, however, that these findings were obtained under isolated testing conditions and did not account for the influence of frictional effects during actual motor operation. The study demonstrates that the novel preload device can provide a suitable preload range for the π-shaped motor, thereby ensuring its stable operation. Furthermore, the stiffness characteristics of the device satisfy the established design criteria for linear ultrasonic motor preload systems, suggesting its potential for enhancing the output performance and operational stability of the π-shaped motor.

## 4. Novel Motion Platform

### 4.1. Structure of Motion Platform

Linear ultrasonic motors exhibit rapid response, high positioning accuracy, and direct linear motion output without requiring transmission mechanisms. Consequently, motion platforms driven by these motors feature short transmission chains, large strokes, and high positioning accuracy. A typical platform configuration comprises a stator, mover, clamping device, and base, with the mover typically supported by commercial linear guide rails. Such rails offer stable performance and high positioning accuracy, making them well-suited for mover applications. This paper presents a one-dimensional motion platform design for a π-shaped stator. As illustrated in Figure 4, two THK VRT 2095A linear guide rails serve as the mover, interconnected by a mounting plate. The platform dimensions are 174 mm × 176 mm × 42 mm with a maximum stroke of 60 mm. This stroke length is constrained by the selected guide rails and can be adjusted by substituting rails of different travel lengths.

However, as commercially mature components, these commercial components exhibit inherent volumetric constraints that limit platform miniaturization. The VRT 2095A rail adopted in this study exemplifies this limitation: while providing a 60 mm maximum stroke, its overall footprint reaches 95 mm × 30 mm × 12 mm due to the combined bulk of rail/slider assemblies and lubrication grooves. To overcome these spatial restrictions while extending effective stroke length, we propose a novel mover design utilizing ceramic bars instead of conventional rails. Although ceramic–ceramic contact modes have been documented, their implementation in linear ultrasonic motor-driven motion platforms has not been extensively explored. This ceramic-based architecture significantly reduces platform volume, increases stroke capacity, and leverages inherent self-lubricating properties that eliminate external lubrication requirements.

**Figure 11:**
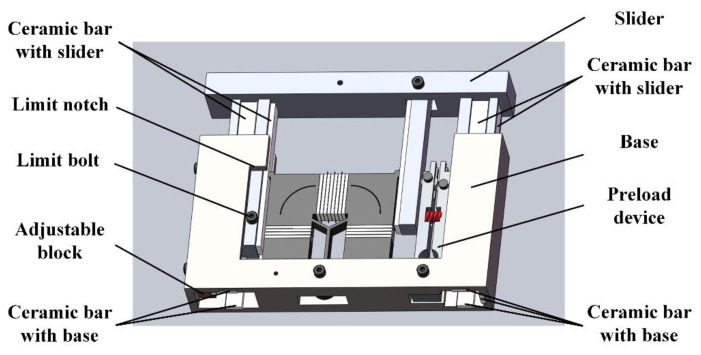
Specific view of new motion platform.

Figure 11 presents the novel motion platform configuration, comprising a base, stator, mover, and preload device. The stator and preload device are rigidly mounted on the base, while six paired ceramic bars bonded to the mover and base surfaces establish exclusive contact interfaces between these components. These bars protrude above adjacent surfaces to ensure isolated ceramic-on-ceramic contact. A mechanical limit mechanism is implemented through a notched slot on the base and a corresponding bolt on the mover, with the notch length dictating maximum travel distance.

Utilizing 100 mm long ceramic bars, the platform achieves dimensions of 174 mm × 107 mm × 28 mm at 60 mm stroke. Compared to linear-guide platforms, this design maintains identical length but reduces width and height to 60% and 67%, respectively. Stroke capacity is extendable to 90 mm, demonstrating superior compactness: equivalent strokes yield 40% smaller volume, while fixed volumes enable 50% greater operating ranges.

### 4.2. Experiments on Motion Platform

**Figure 12:**
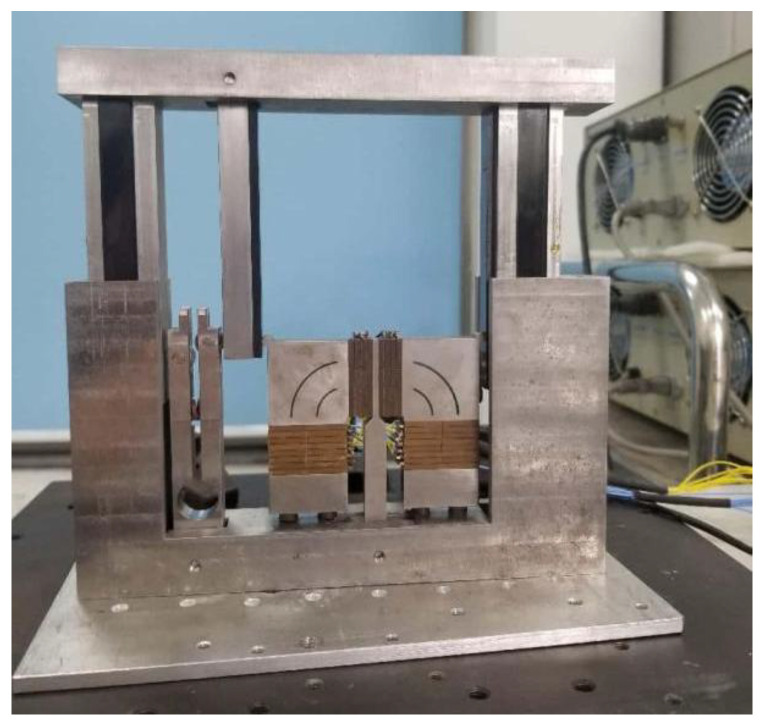
Prototype for platform.

Based on the design solution detailed above, a prototype of the motion platform was fabricated and assembled, as illustrated in Figure 12.

**Figure 13:**
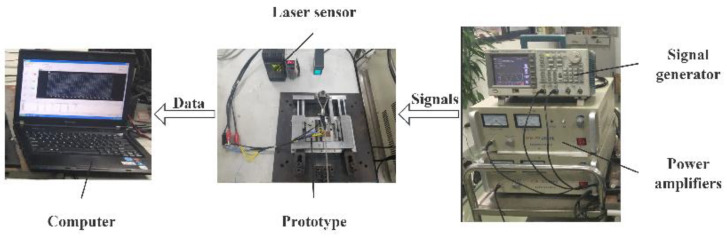
Experimental setup.

To characterize the output performance of the novel platform, the experimental system depicted in Figure 13 was established. A signal generator (AFG3022B, Tektronix, Beaverton, OR, USA) and two power amplifiers (HFVP-153, Foneng, Nanjing, China) serve as the driving signal source, supplying the two-phase sinusoidal excitation signals required for motor operation. During testing, the motor is mounted on the test platform with the laser displacement sensor (LK-H150, Keyence Corp., Osaka, Japan) probe positioned at an appropriate location on the platform. This “appropriate location” is defined such that (1) the mover’s stroke remains within the sensor’s measurement range throughout motor operation, and (2) the sensor’s measurement point maintains a stationary position relative to the mover. This positioning ensures measurement accuracy. Upon test initiation, excitation signals are applied to the stator to activate the motor, driving the mover. The laser displacement sensor records the mover’s displacement and velocity, transmitting this data to a computer which subsequently generates corresponding displacement and velocity profiles.

**Figure 14:**
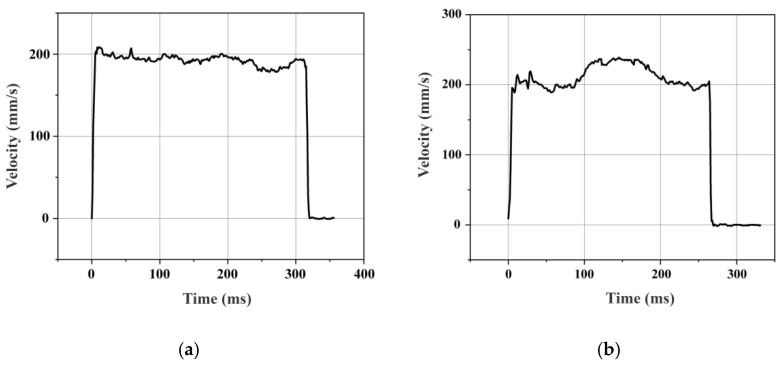
Velocity response curve of motion platform. (**a**) Retraction mode. (**b**) Extension mode.

**Figure 15:**
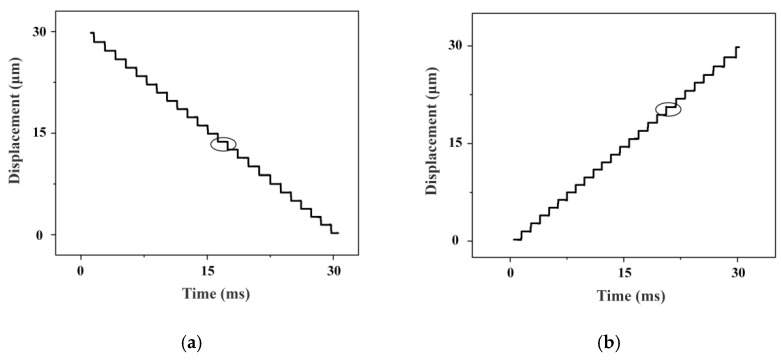
Displacement resolution of motion platform. (**a**) Retraction mode. (**b**) Extension mode.

The fundamental performance of the motion platform was evaluated, focusing on its operating velocity and displacement resolution. The velocity response curves for a 60 mm stroke are presented in Figure 14. The platform exhibits a rapid response, reaching steady state within 10 ms. In the extension direction, a peak velocity of 248 mm/s was attained, completing the full stroke in 270 ms. Conversely, in the retraction direction, the peak velocity was 208 mm/s, with the stroke completed in 320 ms. This measurable difference in velocity between directions is attributed to inherent operational asymmetries in the motor. Furthermore, velocity fluctuations during operation are observed, primarily attributable to surface roughness at the ceramic contact interface and tolerances from machining and assembly. The displacement resolution was also characterized. Using a short 30 ms driving pulse, the minimum incremental displacement in both directions was measured to be 1.1 μm, as shown in Figure 15.

## 5. Conclusions

This paper proposes a novel motion platform driven by a π-shaped linear ultrasonic motor. The design addresses key limitations in original platform, particularly the issue of preload instability during operation. In accordance with design criteria for preloading mechanisms, a stationary preload device was developed and mounted on the base. This configuration ensures that the preload device remains decoupled from the stator’s high-frequency vibrations and does not undergo displacement with the mover. As a result, a consistent preload force is maintained throughout the entire operational cycle, significantly improving the platform’s stability and positioning accuracy. Furthermore, a ceramic-based mover was designed to replace traditional linear guides. This innovation not only reduces the overall platform volume to 40% of that of conventional designs but also extends the travel range to 150% of the original capacity. A prototype was fabricated to experimentally validate the proposed design. Experimental results confirm that the novel motion platform achieves both miniaturization and extended stroke while retaining satisfactory output performance and positioning precision.

## Figures and Tables

**Table 1:** Dimensions of preload device.

Parameters	*a*	*b*	*c*	*l*_1_	*R*	*t*_α_
Values	17	10	96	30	6	2.5

## Data Availability

The original contributions presented in the study are included in the article, further inquiries can be directed to the corresponding author.

## References

[1] Du P., Chen W., Deng J., Zhang S., Zhang J., Liu Y. (2023). A critical review of piezoelectric ultrasonic transducers for ultrasonic-assisted precision machining. Ultrasonics.

[2] Ghafarian M., Shirinzadeh B., Al-Jodah A., Das T.K., Wei W., Tian Y., Zhang D. (2020). An XYZ micromanipulator for precise positioning applications. J. Micro-Bio Robot..

[3] Bansevicius R., Mazeika D., Jurenas V., Kulvietis G., Drukteiniene A., Wallaschek J. (2019). Multi-DOF Ultrasonic Actuators for Laser Beam Positioning. Shock Vib..

[4] Wang R., Wu H., Wang H., Zhang X. (2020). Design and stiffness modeling of a four-degree-of-freedom nanopositioning stage based on six-branched-chain compliant parallel mechanisms. Rev. Sci. Instrum..

[5] Zhao C. (2011). Ultrasonic Motors: Technologies and Applications.

[6] Watson B., Friend J., Yeo L. (2009). Piezoelectric ultrasonic micro/milli-scale actuators. Sens. Actuators A Phys..

[7] Tian X., Liu Y., Deng J., Wang L., Chen W. (2020). A review on piezoelectric ultrasonic motors for the past decade: Classification, operating principle, performance, and future work perspectives. Sens. Actuators A Phys..

[8] Mohith S., Upadhya A.R., Navin K.P., Kulkarni S.M., Rao M. (2020). Recent trends in piezoelectric actuators for precision motion and their applications: A review. Smart Mater. Struct..

[9] Zhu W.L., Zhu Z., Shi Y., Wang X., Guan K., Ju B.-F. (2016). Design, modeling, analysis and testing of a novel piezo-actuated XY compliant mechanism for large workspace nano-positioning. Smart Mater. Struct..

[10] Li J., Huang H., Morita T. (2019). Stepping piezoelectric actuators with large working stroke for nano-positioning systems: A review. Sens. Actuators A Phys..

[11] Liu Z., Fu Q., Yang P., Dong Z., Zhang L., Yao Z. (2023). Design and performance evaluation of a miniature I-shaped linear ultrasonic motor with two vibrators. Ultrasonics.

[12] Ma X., Liu J., Deng J., Liu Q., Liu Y. (2020). A Rotary Traveling Wave Ultrasonic Motor With Four Groups of Nested PZT Ceramics: Design and Performance Evaluation. IEEE Trans. Ultrason. Ferroelectr. Freq. Control.

[13] Li H., Liu Y., Deng J., Chen W., Li K. (2021). Design Philosophy for Ultrasonic Motors Using the Bending Hybrid Modes. Sens. Actuators A Phys..

[14] Wang L., Wang J.A., Jin J.M., Yang L., Wu S.-W., Zhou C.C. (2022). Theoretical modeling, verification, and application study on a novel bending-bending coupled piezoelectric ultrasonic transducer. Mech. Syst. Signal Process..

[15] Jian Y., Liu Z., He J., Zhou W., Liang H. (2024). Development of a Plate Linear Ultrasonic Motor Using the Power Flow Method. Micromachines.

[16] Kuribayashi Kurosawa M., Kodaira O., Tsuchitoi Y., Higuchi T. (1998). Transducer for high speed and large thrust ultrasonic linear motor using two sandwich-type vibrators. IEEE Trans. Ultrason. Ferroelectr. Freq. Control.

[17] Li X., Yao Z. (2016). Analytical modeling and experimental validation of a V-shape piezoelectric ultrasonic transducer. Smart Mater. Struct..

[18] Asumi K., Fukunaga R., Fujimura T., Kurosawa M.K. (2009). High speed, high resolution ultrasonic linear motor using V-shape two bolt-clamped Langevin-type transducers. Acoust. Sci. Technol..

[19] Asumi K., Fukunaga R., Fujimura T., Kurosawa M.K. (2009). Miniaturization of a V-Shape Transducer Ultrasonic Motor. Jpn. J. Appl. Phys..

[20] Jeong S.S., Park T.G., Kim M.H., Song T.-K. (2011). Characteristics of a V-type ultrasonic rotary motor. Curr. Appl. Phys..

[21] Zhang H., Dong S.X., Zhang S.Y., Wang T.-H., Zhang Z.-N., Fan L. (2006). Ultrasonic micro-motor using miniature piezoelectric tube with diameter of 1.0 mm. Ultrasonics.

[22] Takano M., Takimoto M., Nakamura K. (2011). Electrode design of multilayered piezoelectric transducers for longitudinal-bending ultrasonic actuators. Acoust. Sci. Technol..

[23] Xin X., Gao X., Wu J., Li Z., Chu Z., Dong S. (2020). A ring-shaped, linear piezoelectric ultrasonic motor operating in *E_01_* mode. Appl. Phys. Lett..

[24] Guo X., Zhang Y., Jin D., Zhou M. (2022). A Review of Single-Cell Pose Adjustment and Puncture. Adv. Intell. Syst..

[25] Masuda N., Izuhara S., Mashimo T. (2023). Miniature camera module with a hollow linear ultrasonic motor-based focus feature. Sens. Actuators A Phys..

[26] Tu Y., Peng F., Wilson D.A. (2017). Motion Manipulation of Micro- and Nanomotors. Adv. Mater..

[27] Zhu Z., To S., Ehmann K.F., Zhou X. (2017). Design, analysis, and realization of a novel piezoelectrically actuated rotary spatial vibration system for micro-/nanomachining. IEEE/ASME Trans. Mechatron..

[28] Li X., Wang X., Sun W., Wang K., Yang Z., Liang T., Huang H. (2020). A compact 2-DOF piezo-driven positioning stage designed by using the parasitic motion of flexure hinge mechanism. Smart Mater. Struct..

[29] Li J., Liu H., Zhao H. (2017). A Compact 2-DOF Piezoelectric-Driven Platform Based on “Z-Shaped” Flexure Hinges. Micromachines.

[30] Jian Y., Yao Z., Liu Z., Zhang B. (2018). A novel Π -type linear ultrasonic motor driven by a single mode. Rev. Sci. Instrum..

[31] Jian Y., Yao Z., Silberschmidt V.V. (2017). Linear Ultrasonic Motor for Absolute Gravimeter. Ultrasonics.

[32] Lobontiu N., Garcia E. (2003). Analytical model of displacement amplification and stiffness optimization for a class of flexure-based compliant mechanisms. Comput. Struct..

